# Clinical Features and Potential Mechanisms Relating Neuropathological Biomarkers and Blood-Brain Barrier in Patients With Alzheimer’s Disease and Hearing Loss

**DOI:** 10.3389/fnagi.2022.911028

**Published:** 2022-06-16

**Authors:** Wei-jiao Zhang, Dan-ning Li, Teng-hong Lian, Peng Guo, Ya-nan Zhang, Jing-hui Li, Hui-ying Guan, Ming-yue He, Wen-jing Zhang, Wei-jia Zhang, Dong-mei Luo, Xiao-min Wang, Wei Zhang

**Affiliations:** ^1^Department of Neurology, Beijing Tiantan Hospital, Capital Medical University, Beijing, China; ^2^Center for Cognitive Neurology, Department of Neurology, Beijing Tiantan Hospital, Capital Medical University, Beijing, China; ^3^Department of Blood Transfusion, Beijing Tiantan Hospital, Capital Medical University, Beijing, China; ^4^Department of Physiology, Capital Medical University, Beijing, China; ^5^China National Clinical Research Center for Neurological Diseases, Beijing Tiantan Hospital, Capital Medical University, Beijing, China; ^6^Center of Parkinson’s Disease, Beijing Institute for Brain Disorders, Beijing, China; ^7^Beijing Key Laboratory on Parkinson’s Disease, Beijing, China

**Keywords:** Alzheimer’s disease, hearing loss, clinical features, neuropathological biomarkers of AD, blood-brain barrier, cerebrospinal fluid

## Abstract

**Background:**

The aim of this study was to explore clinical features and potential mechanisms relating neuropathological biomarkers and blood-brain barrier (BBB) in Alzheimer’s disease (AD) and hearing loss (HL).

**Materials and Methods:**

A total of 65 patients with AD were recruited and auditory function was assessed by threshold of pure tone audiometry (PTA). Patients were divided into AD with HL (AD-HL) and AD with no HL (AD-nHL) groups based on the standard of World Health Organization. Clinical symptoms were assessed by multiple rating scales. The levels of neuropathological biomarkers of β amyloid1-42 (Aβ_1–42_) and multiple phosphorylated tau (P-tau), and BBB factors of matrix metalloproteinases (MMPs), receptor of advanced glycation end products, glial fibrillary acidic protein, and low-density lipoprotein receptor related protein 1 were measured.

**Results:**

(1) Compared with AD-nHL group, AD-HL group had significantly impaired overall cognitive function and cognitive domains of memory, language, attention, execution, and activities of daily living (ADL) reflected by the scores of rating scales (*P* < 0.05). PTA threshold was significantly correlated with the impairments of overall cognitive function and cognitive domains of memory and language, and ADL in patients with AD (*P* < 0.05). (2) P-tau (S199) level was significantly increased in CSF from AD-HL group (*P* < 0.05), and was significantly and positively correlated with PTA threshold in patients with AD. (3) MMP-3 level was significantly elevated in CSF from AD-HL group (*P* < 0.05), and was significantly and positively correlated with PTA threshold in patients with AD (*P* < 0.05). (4) In AD-HL group, P-tau (S199) level was significantly and positively correlated with the levels of MMP-2 and MMP-3 in CSF (*P* < 0.05).

**Conclusion:**

AD-HL patients have severely compromised overall cognitive function, multiple cognitive domains, and ADL. The potential mechanisms of AD-HL involve elevations of AD neuropathological biomarker of P-tau (S199) and BBB factor of MMP-3, and close correlations between P-tau (S199) and MMP-2/MMP-3 in CSF. Findings from this investigation highly suggest significance of early evaluation of HL for delaying AD progression, and indicate new directions of drug development by inhibiting neuropathological biomarkers of AD and protecting BBB.

## Introduction

Alzheimer’s disease (AD) is the most common form of neurodegenerative disease and ranks the top among all cognitive disorders, contributing to approximately 60–70% of the total cases worldwide (World Health Organization, 2020). The recently published epidemiological data from China showed that 9.83 million of patients with AD aged 60 years or older out of 15.07 million dementia cases (Jia et al., 2020). The neuropathological features of AD include neuritic plaques and neurofibrillary tangles with β amyloid (Aβ) and hyperphosphorylated tau (P-tau) as major components, respectively. AD usually starts insidiously and worsens progressively with clinical symptoms of cognitive impairment, neuropsychiatric symptoms, and compromised activities of daily living (ADL), which brings about heavy economic and caregiver burdens to both families and society.

Hearing loss (HL) is one of the most common symptoms in the elderly population and its prevalence is increasing with age. In the population older than 60 years, over 25% of the total population were affected by disabling HL (World Health Organization, 2021). In the meta-analysis of prospective cohort studies, HL patients had the relative risk of 2.82 of developing mild cognitive impairment (MCI) due to AD and AD dementia, and 4.87 of developing AD dementia (Zheng et al., 2017). Additionally, it was found that hearing aids was helpful in delaying or preventing cognitive decline (Taljaard et al., 2016; Maharani et al., 2018). Hence, HL is one of the modifiable risk factors of AD.

Currently, there are insufficient studies on the clinical features of AD with HL (AD-HL). It was found that AD mice with HL had significantly impaired memory (Kim et al., 2020). However, there is no investigation about the frequency and clinical features of AD patients with HL.

Increasing evidence showed that sensory abnormalities, such as HL (Kim et al., 2020), olfactory (Kim et al., 2020), and visual dysfunctions (Hart et al., 2016) were related to AD. Accordingly, depositions of neuropathological biomarkers of AD, Aβ, and P-tau in the sensory organs have attracted widespread attention. Autopsy studies from AD patients demonstrated that tau pathology in the olfactory epithelium and areas was related to olfactory information processing (Attems and Jellinger, 2006; Murphy, 2019). Aβ and P-tau were found in the retina of AD patients (Chiasseu et al., 2017). However, there is no study reporting AD pathology in peripheral auditory organs, such as cochlea. A previous study reported that central auditory processing reflected by dichotic sentence identification (DSI) right ear advantage (REA, right minus left ear score) was correlated with P-tau and total tau (T-tau) but not Aβ in elderly with normal cognition and family history of AD (Tuwaig et al., 2017). However, few studies pay attention to peripheral auditory processing and its relationship with Aβ and multiple forms of P-tau in AD patients.

Blood-brain barrier (BBB) is a protective structure for brain, and it’s damage can be reflected by the alterations of related factors, including matrix metalloproteinases (MMPs), receptor of advanced glycation end products (RAGE), glial fibrillary acidic protein (GFAP), and low-density lipoprotein receptor related protein 1 (LRP1). In animal experiment on AD and HL, the elevations of MMPs indicated impairment of BBB (Wang et al., 2014; Wu et al., 2017). In patients with AD, MMPs levels were significantly elevated (Wu et al., 2017). For examples, MMP-9 was observed in neuritic plaques, neurofibrillary tangles, cytoplasm of neurons, and vascular walls in hippocampus and cortex; MMP-9 level was even significantly elevated in serum from patients with MCI due to AD compared with control (Lorenzl et al., 2003, 2008). Meanwhile, MMP-9 level was also significantly elevated in cochlea from HL patients (Wu et al., 2017). However, there is no investigation about the relationship between AD-HL and BBB factors.

At present, there is no study on the relationship between the clinical features and potential mechanisms relating neuropathological biomarkers of AD and BBB factors in AD-HL patients. Hence, in this study, demographic variables of patients with AD were collected, and a host of professional rating scales were used to evaluate cognitive impairment, neuropsychiatric symptoms, and ADL. Pure tone audiometry (PTA) threshold was detected by otorhinolaryngology doctors. The levels of neuropathological biomarkers of AD, including Aβ_1–42_, P-tau (T181), P-tau (S199), P-tau (T231), P-tau (S396), and T-tau, and BBB factors, including MMP-2, MMP-3, MMP-9, RAGE, GFAP, and LRP1 in cerebrospinal fluid (CSF) from patients with AD were measured by enzyme-linked immunosorbent assay (ELISA). The above variables were compared between AD-HL and AD with no HL (AD-nHL) groups, and the correlations among the above variables were analyzed in patients with AD. Results from this study may help understand the clinical features and potential mechanisms of AD-HL, indicate the significance of early identification of HL, and provide potential therapeutic target for the drug development of AD.

## Materials and Methods

### Ethics Statement

This study was approved by the Review Board of Beijing Tiantan Hospital, Capital Medical University, and written informed consents were obtained from all participants and their caregivers.

### Participants

#### Inclusion Criteria

This study included patients with MCI due to AD and patients with AD dementia according to the National Institute of Aging and Alzheimer’s Association (NIA-AA) criteria (Albert et al., 2011; McKhann et al., 2011).

#### Exclusion Criteria

The exclusion criteria of this study were as follows: (1) the presence of neurological disorders besides AD that might affect cognition, including Parkinson’s disease, multiple sclerosis, epilepsy, etc.; (2) the presence of systemic diseases, including hypothyroidism, severe chronic diseases, and other medical diseases that might affect hearing; (3) histories of alcoholism, carbon monoxide poisoning, etc.; (4) the presence of one or more of following otological diseases, including acoustic nerve dysplasia, common cavity and other serious inner ear malformations, acoustic neuropathy, and acoustic neuroma; and (5) the inability to cooperate with subjective speech tests.

### Detection of Pure Tone Audiometry Threshold

PTA was performed by the doctors using an audiometer (Astera, Conera TM) in a sound-isolated room in the Department of Otorhinolaryngology in our hospital.

PTA threshold refers to the average of hearing level at a set of specified frequencies, which gives a snapshot of hearing level of each ear. The frequencies used for PTA were 500, 1,000, 2,000, and 4,000 Hz, with an intensity range of –10 to 110 dB. PTA threshold was the average of intensities measured at the abovementioned frequencies. World Health Organization defined a PTA ≥ 20 dB, in either ear, as HL (World Health Organization, 2021).

### Collections of Demographic Variables

Demographic variables of gender, age, age of onset, disease duration, and years of education were collected.

### Assessment of Cognitive Function

#### Overall Cognitive Function

Mini-Mental State Examination (MMSE) and Montreal Cognitive Assessment (MoCA) scales were used to rate the overall cognitive function of patients with AD (Cockrell and Folstein, 1988; Pinto et al., 2019). Patients with illiteracy, primary education, or more than a junior education were identified as having cognitive impairment when the MMSE score was below 17, 20, or 24 points, respectively. MoCA score ≤ 26 indicated potential cognitive impairment. If the educational level of an individual was less than 12 years, 1 point was added. The lower are the scores of the two scales, the severe is the overall cognitive impairment.

A variety of cognitive domains were assessed by the following rating scales:

##### Memory

Auditory Verbal Learning Test (AVLT) was used to assess verbal memory. AVLT N1-3, AVLT N4, and AVLT N5 evaluated immediate recall, short-delayed recall, and long-delayed recall, respectively. AVLT N1-5 reflected the general state of verbal memory. AVLT N6 tested logical memory and AVLT N7 rated ability of recognition (Guo et al., 2009). The Complex Figure Test (CFT)-delayed memory was used to assess visual delayed memory (Zhou et al., 2006). The lower score of this test suggested worse memory.

##### Visuospatial Ability

Visuospatial ability was evaluated by using the CFT-imitation (Zhou et al., 2006). The lower score of this test indicated worse visuospatial ability.

##### Language

Language function was evaluated by using the Boston Naming Test (BNT) (Lin et al., 2014) and Verbal Fluency Test (VFT), including Animal Fluency Test (AFT), VFT-household items (VFT-H), and VFT-alternating fluency (Sebaldt et al., 2009). The decreased scores of these scales implied compromised language function.

##### Attention

Attention was evaluated by using the Trail Making Test A, B (TMT-A, B) and Symbol Digit Modalities Test (SDMT) (Gong, 1992; Guo et al., 2007). The longer it took to complete the TMT-A and TMT-B, the worse was the attention. The lower TMT-A, TMT-B, and SDMT scores indicated more severe attention deficit.

##### Executive Function

Executive function was rated by using the Stroop Color-Word Test (SCWT) (Guo et al., 2007). The reduced score of this test revealed an impaired executive function.

### Assessment of Neuropsychiatric Symptoms

Overall neuropsychiatric symptoms were assessed by using the Neuropsychiatric Inventory (NPI). The higher score implied severe overall neuropsychiatric symptoms (Wolinsky et al., 2018).

Individual neuropsychiatric symptoms were then assessed by using the following rating scales.

#### Depression

Depression was evaluated by using the Hamilton Depression Scale (HAMD)-24 items. The higher score indicated more severe depression, and a score of ≥ 8 suggested the presence of depression (Whisman et al., 1989).

#### Anxiety

Anxiety was evaluated by using the Hamilton Anxiety Scale (HAMA)-14 items. The higher score suggested more severe anxiety, and a score of ≥8 indicated the existence of anxiety (Guy, 1976).

#### Agitation

Agitation was rated by using the Cohen-Mansfield Agitation Inventory (CMAI). The elevated CMAI score displayed severe agitation (Lin et al., 2007).

#### Apathy

Apathy was rated by using the Modified Apathy Estimate Scale (MAES). The higher was the MAES score, the more severe was the apathy. A score of >14 revealed clinically meaningful apathy (Guercio et al., 2015).

#### Daytime Sleepiness

Daytime sleepiness was assessed by using Epworth Sleepiness Scale (ESS). The higher was the ESS score, the more severe was the daytime sleepiness. A score of >10 implied daytime sleepiness (Lee et al., 2007).

### Assessment of Activities of Daily Living

ADL was assessed by using the ADL scale, which included the basic ADL (BADL) and the instrumental ADL (IADL) scales. The enhanced score of ADL scale demonstrated the compromised ADL performance (Katz et al., 1963).

### Collection and Processing of Cerebrospinal Fluid Samples

Anti-AD drugs were withdrawn for 12–14 h prior to CSF collection. In order to prevent the blood contamination of CSF, the lumber puncture for each patient was performed by a professionally trained neurologist, strictly following the standardized protocol. The first and second tubes might contain blood, as well as tissue fragments and contaminated skin microorganisms; thus, routinely, the third tube of CSF was retained for the measurement. CSF samples from both AD-HL and AD-nHL groups in this study all followed this standardized protocol, trying to avoid this potential bias. A total of 5 ml CSF was taken in a polypropylene tube (Beijing JingkeHongda Biotechnology Co., Ltd.) under a fasting condition.

CSF samples were centrifuged immediately at 2,000 *g* at 4°C. Approximately 0.5 ml CSF supernatant was aliquoted into separate Nunc cryotubes (Beijing JingkeHongda Biotechnology Co., Ltd.) and kept frozen at −80°C until further assay.

### Measurements of Neuropathological Biomarkers of Alzheimer’s Disease in Cerebrospinal Fluid

The levels of neuropathological biomarkers of AD, including Aβ_1–42_, P-tau (T181), P-tau (S199), P-tau (T231), P-tau (S396), and T-tau, in CSF from patients in AD-HL and AD-nHL groups were detected by using ELISA. CSB-E10684h kit and CSBE12011h kit (CUSABIO Company, Wuhan, China) were used for the measurements of Aβ_1–42_ and T-tau, respectively. KHB7031 kit, KHO0631 kit, KHB7041 kit, and KhB8051 kit (Invitrogen Company, Carlsbad, CA, United States) were used for the measurements of P-tau (T181), P-tau (S199), P-tau (T231), and P-tau (S396), respectively.

### Measurements of Blood-Brain Barrier Factors in Cerebrospinal Fluid

The levels of BBB factors, including MMP-2, MMP-3, MMP-9, RAGE, GFAP, and LRP1, in CSF from patients in AD-HL and AD-nHL groups were detected by using ELISA. MMP200 kit, DMP300 kit, DMP900 kit, and DRG00 kit (R&D systems Company, Minneapolis, MN, United States) were used for the measurements of MMP-2, MMP-3, MMP-9, and RAGE, respectively. NS830 kit (Merck Millipore Company, Darmstadt, Germany) was used for the measurement of GFAP. MBS772326 kit (MyBioSource Company, San Diego, CA, United States) was used for the measurement of LRP1.

### Data Analyses

Statistical analyses were performed by using the SPSS Statistics 23.0 (IBM Corp., Armonk, NY, United States). A value of *P* < 0.05 was considered statistically significant. Continuous variables, if normally distributed, were presented as means ± SDs, and were compared by using the two-sample *t*-test. Continuous variables, if they were not normally distributed, were presented as medians (quartiles), and were compared by using a non-parametric test. The bivariate correlation method was used to analyze the relationships among the variables.

## Results

### The Frequency of Alzheimer’s Disease With Hearing Loss

Among the 65 patients with AD who were analyzed, 21 cases (32.31%) had no HL, and 44 cases had HL with the frequency up to 67.69%, indicating that HL was very common in AD patients. Among the AD patients with HL, 25 cases (38.46%) had mild HL, 14 cases (21.54%) had moderate HL, 5 cases (7.69%) had moderate to severe HL, and 0 cases (0.00%) had severe HL.

### Demographic Variables of Alzheimer’s Disease With No Hearing Loss and Alzheimer’s Disease With Hearing Loss Groups

Demographic variables, including sex, age, age of onset, disease duration, and years of education were compared between AD-nHL and AD-HL groups (Table 1). The results showed that demographic variables had no significant differences between the two groups.

**TABLE 1:** Demographic variables of Alzheimer’s disease with no hearing loss (AD-nHL) and Alzheimer’s disease with hearing loss (AD-HL) groups.

Demographic variables	AD-nHL group	AD-HL group	*P*
	(21 cases)	(44 cases)	
Female (n, %)	15 (71.43%)	22 (50.00%)	0.105
Age [year, Median (Q1–Q3)]	61.00 (56.50–62.50)	64.00 (57.00–69.00)	0.103
Age of onset [year, Median (Q1–Q3)]	57.00 (55.00–61.00)	61.00 (53.25–65.00)	0.246
Disease duration [year, Median (Q1–Q3)]	36.00 (22.00–53.50)	35.50 (12.00–57.00)	0.557
Years of education [year, Median (Q1-Q3)]	11.00 (9.00–14.25)	9.00 (9.00–12.00)	0.318

### Cognitive Function, Neuropsychiatry Symptoms, and Activities of Daily Living in Alzheimer’s Disease With No Hearing Loss and Alzheimer’s Disease With Hearing Loss Groups

The scores of cognitive functions, neuropsychiatry symptoms, and ADL were compared between AD-nHL and AD-HL groups (Table 2). The data displayed that AD-HL group had a significantly lower score of MMSE and MoCA scales, and scores of AVLT, VFT, BNT, TMT-B, SCWT-C, and SDMT than AD-nHL group. There were no significant differences in neuropsychiatry symptoms between the two groups. AD-HL group had a significantly higher ADL score than AD-nHL group (*P* < 0.05).

**TABLE 2:** Cognitive function, neuropsychiatry symptoms, and activities of daily living (ADL) between AD-nHL and AD-HL groups.

	AD-nHL group	AD-HL group	*P*
	(21 cases)	(44 cases)	
**Cognitive function**			
MMSE (point, x̄ ± s)	20.24 ± 7.19	15.36 ± 8.53	0.032*
MoCA (point, x̄ ± s)	14.76 ± 6.66	10.88 ± 7.39	0.048*
AVLT N1-3 (point, x̄ ± s)	12.68 ± 5.84	8.00 ± 5.65	0.005**
AVLT N4 [point, Median (Q1–Q3)]	2.50 (0.00–4.50)	0.00 (0.00–3.00)	0.055
AVLT N5 [point, Median (Q1–Q3)]	2.00 (0.00–5.00)	0.00 (0.00–2.25)	0.010*
AVLT N1-5 [point, Median (Q1–Q3)]	15.00 (7.00–26.00)	8.00 (0.25–14.75)	0.017*
AVLT N6 [point, Median (Q1–Q3)]	2.00 (0.00–6.00)	0.00 (0.00–2.00)	0.009**
AVLT N7 [point, Median (Q1–Q3)]	9.00 (7.50–13.00)	3.00 (0.00–11.00)	0.019*
AFT (point, x̄ ± s)	14.67 ± 4.91	9.84 ± 5.34	0.001**
VFT-H (point, x̄ ± s)	13.47 ± 4.62	8.52 ± 5.09	<0.001**
VFT-alternating fluency[point, Median (Q1–Q3)]	11.00 (7.00–14.00)	5.00 (1.00–9.25)	0.001**
BNT [point, Median (Q1–Q3)]	24.50 (19.00–25.75)	20.00 (8.00–23.00)	0.012*
TMT-A [point, Median (Q1–Q3)]	25.00 (23.00–25.00)	25.00 (10.00–25.00)	0.355
TMT-B [point, Median (Q1–Q3)]	23.00 (16.00–25.00)	16.00 (0.00–22.75)	0.014*
CFT-imitation [point, Median (Q1–Q3)]	31.50 (22.88–34.25)	30.00 (0.00–34.50)	0.221
CFT-delayed [point, Median (Q1–Q3)]	5.00 (0.00–12.00)	0.00 (0.00–19.00)	0.374
SCWT-A [point, Median (Q1–Q3)]	50.00 (48.50–50.00)	50.00 (38.75–50.00)	0.344
SCWT-B [point, Median (Q1–Q3)]	50.00 (48.74–50.00)	48.50 (44.75–50.00)	0.083
SCWT-C [point, Median (Q1–Q3)]	48.50 (40.75–50.00)	40.00 (1.00–47.00)	0.010*
SDMT [point, Median (Q1–Q3)]	31.00 (15.00–39.50)	14.00 (0.00–22.25)	0.007**
**Neuropsychiatry symptoms**			
HAMD [point, Median (Q1–Q3)]	7.00 (3.50–12.75)	5.00 (4.00–10.00)	0.390
HAMA [point, Median (Q1–Q3)]	6.00 (2.25–11.75)	6.00 (3.00–8.00)	0.841
NPI [point, Median (Q1–Q3)]	2.50 (0.00–7.75)	2.00 (0.00–11.75)	0.723
MEAS (point, x̄ ± s)	14.81 ± 9.30	16.00 ± 10.09	0.655
CMAI [point, Median (Q1–Q3)]	29.00 (29.00–30.00)	29.00 (29.00–31.00)	0.848
ESS [point, Median (Q1–Q3)]	3.00 (1.00–5.00)	2.00 (0.00–6.75)	0.866
**ADL**			
ADL [point, Median (Q1–Q3)]	20.00 (20.00–25.50)	24.00 (20.00–38.00)	0.038*

*P < 0.05, **P < 0.01. The bold values are statistically significant.

### Correlation of Pure Tone Audiometry Threshold With the Scores of Clinical Symptoms in Alzheimer’s Disease Patients

The correlations of PTA threshold with the scores of clinical symptoms in AD patients were analyzed (Table 3). The results suggested that PTA threshold was significantly and negatively correlated with the scores of MMSE, AVLT N1-3, N1-5, N7, VFT, and BNT in AD patients (*P* < 0.05), and was significantly and positively correlated with the score of ADL (*P* < 0.05).

**TABLE 3:** Correlations of pure tone audiometry (PTA) threshold with the scores of clinical symptoms from Alzheimer’s disease (AD) patients.

	*R*	*P*
MMSE (point, x̄ ± s)	**−0.287**	0.031*
MoCA (point, x̄ ± s)	**−**0.232	0.072
AVLT N1–3 (point, x̄ ± s)	**−0.334**	**0.010** *
AVLT N4 [point, Median (Q1–Q3)]	**−**0.204	0.151
AVLT N5 [point, Median (Q1–Q3)]	**−**0.081	0.550
AVLT N1**–**5 [point, Median (Q1–Q3)]	**−0.301**	0.015*
AVLT N6 [point, Median (Q1–Q3)]	**−**0.226	0.115
AVLT N7 [point, Median (Q1–Q3)]	**−0.373**	**0.008** **
AFT (point, x̄ ± s)	**−0.315**	0.018*
VFT-H (point, x̄ ± s)	**−0.415**	**0.001** **
VFT-alternating fluency [point, Median (Q1–Q3)]	**−0.370**	**0.005** **
BNT [point, Median (Q1–Q3)]	**−0.395**	**0.003** **
TMT-A [point, Median (Q1–Q3)]	**−**0.131	0.354
TMT-B [point, Median (Q1–Q3)]	**−**0.137	0.339
CFT-imitation [point, Median (Q1–Q3)]	**−**0.059	0.683
CFT-delayed [point, Median (Q1–Q3)]	0.186	0.211
SCWT-A [point, Median (Q1–Q3)]	**−**0.222	0.113
SCWT-B [point, Median (Q1–Q3)]	**−**0.244	0.082
SCWT-C [point, Median (Q1–Q3)]	**−**0.253	0.076
SDMT [point, Median (Q1–Q3)]	**−**0.258	0.080
ADL [point, Median (Q1–Q3)]	**0.247**	**0.049** *

*P < 0.05, **P < 0.01. The bold values are statistically significant.

### Levels of Neuropathological Biomarkers in Cerebrospinal Fluid From Alzheimer’s Disease With No Hearing Loss and Alzheimer’s Disease With Hearing Loss Groups

The levels of neuropathological biomarkers of AD, including Aβ_1–42_, P-tau (T181), P-tau (S199), P-tau (T231), and P-tau (S396) and T-tau in CSF from AD-nHL and AD-HL groups were compared (Table 4). The results showed that the level of P-tau (S199) was significantly increased in CSF from AD-HL group (*P* < 0.05).

**TABLE 4:** Levels of neuropathological biomarkers of AD in cerebrospinal fluid (CSF) from AD-nHL and AD-HL groups.

	AD-nHL group	AD-HL group	*P*
	(21 cases)	(44 cases)	
Aβ_1–42_ (ng/ml, x̄ ± s)	2.53 ± 2.98	2.48 ± 3.14	0.854
P-tau (T181) (pg/ml, x̄ ± s)	67.02 ± 22.30	68.51 ± 25.84	0.899
P-tau (S199) [pg/ml, Median (Q1–Q3)]	6.54 (4.72–9.02)	8.19 (7.27–14.14)	0.013*
P-tau (T231) (pg/ml, x̄ ± s)	87.95 ± 29.09	85.62 ± 25.78	0.877
P-tau (S396) (pg/ml, x̄ ± s)	75.91 ± 27.24	72.54 ± 24.06	0.682
T-tau (pg/ml, x̄ ± s)	73.45 ± 32.35	85.85 ± 26.74	0.17

*P < 0.05. The bold value is statistically significant.

### Correlation of Pure Tone Audiometry Threshold With the Levels of Neuropathological Biomarkers in Cerebrospinal Fluid From Alzheimer’s Disease Patients

The correlations of PTA threshold with the levels of Aβ_1–42_, P-tau (T181), P-tau (S199), P-tau (T231), P-tau (S396), and T-tau in CSF from AD patients were analyzed (Table 5). It was found that the PTA threshold was significantly and positively correlated with P-tau (S199) level in CSF from AD patients (*P* < 0.05). PTA threshold was not correlated with the levels of Aβ_1–42_, P-tau (T181), P-tau (T231), P-tau (S396), and T-tau in CSF in AD patients (*P* > 0.05).

**TABLE 5:** Correlations of PTA threshold with the levels of neuropathological biomarkers of AD in CSF from AD patients.

	*R*	*P*
Aβ_1–42_ (ng/ml, x̄ ± s)	0.228	0.152
P-tau (T181) (pg/ml, x̄ ± s)	0.291	0.065
P-tau (S199) [pg/ml, Median (Q1–Q3)]	**0.469**	0.002**
P-tau (T231) (pg/ml, x̄ ± s)	0.127	0.430
P-tau (S396) (pg/ml, x̄ ± s)	0.079	0.622
T-tau (pg/ml, x̄ ± s)	0.233	0.107

**P < 0.01. The bold values are statistically significant.

### Levels of Blood-Brain Barrier Factors in Cerebrospinal Fluid From Alzheimer’s Disease With No Hearing Loss and Alzheimer’s Disease With Hearing Loss Groups

The levels of BBB factors, including MMP-2, MMP-3, MMP-9, RAGE, GFAP, and LRP1, in CSF from AD-nHL and AD-HL groups were compared (Table 6). The results indicated that MMP-3 level was significantly elevated in CSF from AD-HL group compared with that from AD-nHL group (*P* < 0.05).

**TABLE 6:** Levels of blood-brain barrier (BBB) factors in CSF from AD-nHL and AD-HL groups.

	AD-nHL group	AD-HL group	*P*
	(21 cases)	(44 cases)	
MMP-2 [ng/ml, Median (Q1–Q3)]	5.17 (2.93–6.52)	7.57 (4.15–9.44)	0.134
MMP-3 [ng/ml, Median (Q1–Q3)]	0.37 (0.31–0.46)	0.52 (0.44–0.89)	0.004**
MMP-9 [ng/ml, Median (Q1–Q3)]	5.85 (5.05–7.69)	6.49 (5.53–7.93)	0.703
RAGE [ng/ml, Median (Q1–Q3)]	2.28 (1.56–4.42)	2.69 (1.45–3.10)	0.923
GFAP [ng/ml, Median (Q1–Q3)]	0.11 (0.07–0.16)	0.09 (0.07–0.11)	0.288
LRP1 [ng/ml, Median (Q1–Q3)]	33.12 (24.52–40.90)	31.82 (25.00–42.01)	0.668

**P < 0.01. The bold value is statistically significant.

### Correlation of Pure Tone Audiometry Threshold With the Levels of Blood-Brain Barrier Factors in Cerebrospinal Fluid From Alzheimer’s Disease Patients

The correlations of PTA threshold with the levels of BBB factors, including MMP-2, MMP-3, MMP-9, RAGE, GFAP, and LRP1, in CSF from AD patients were analyzed (Table 7). It was found that PTA threshold had a significant and positive correlation with MMP-3 level in CSF from AD patients (*P* < 0.05). PTA threshold was not correlated with the levels of MMP 2, MMP 9, RAGE, GFAP, and LRP1 in CSF from AD patients (*P* > 0.05).

**TABLE 7:** Correlations of PTA threshold with the levels of BBB factors in CSF from AD patients.

	*R*	*P*
MMP-2 [ng/ml, Median (Q1–Q3)]	0.254	0.109
MMP-3 [ng/ml, Median (Q1–Q3)]	**0.553**	<0.001**
MMP-9 [ng/ml, Median (Q1–Q3)]	0.069	0.666
RAGE [ng/ml, Median (Q1–Q3)]	<0.001	0.999
GFAP [ng/ml, Median (Q1–Q3)]	**−**0.022	0.892
LRP1 [ng/ml, Median (Q1–Q3)]	0.137	0.369

**P < 0.01. The bold values are statistically significant.

### Correlations of the Levels of Neuropathological Biomarkers With the Levels of Blood-Brain Barrier Factors in Cerebrospinal Fluid From Alzheimer’s Disease With Hearing Loss Patients

The correlations of the levels of neuropathological biomarkers of AD with the levels of BBB factors in CSF from AD-HL patients were analyzed (Table 8). In AD-HL patients, it was found that the P-tau (S199) level had a significant and positive correlation with the levels of MMP-2 and MMP-3 (*P* < 0.05).

**TABLE 8:** Correlations of levels of neuropathological biomarkers of AD with the levels of BBB factors in CSF from AD-HL patients.

	MMP-2		MMP-3		MMP-9		RAGE		GFAP		LRP1	
	*R*	*P*	*R*	*P*	*R*	*P*	*R*	*P*	*R*	*P*	*R*	*P*
Aβ_1–42_ (ng/ml, x̄ ± s)	0.184	0.25	0.122	0.448	0.118	0.462	**−**0.019	0.905	**−**0.105	0.513	0.163	0.328
P-tau (T181) (pg/ml, x̄ ± s)	0.179	0.264	0.196	0.219	0.099	0.538	**−**0.005	0.973	**−**0.01	0.953	**−**0.19	0.252
P-tau (S199) [pg/ml, Median (Q1–Q3)]	**0.419**	**0.006** **	**0.573**	**<0.001** **	0.214	0.179	0.166	0.299	**−**0.118	0.461	0.161	0.328
P-tau (T231) (pg/ml, x̄ ± s)	0.162	0.311	0.155	0.334	**−**0.056	0.728	**−**0.125	0.436	0.075	0.641	0.294	0.073
P-tau (S396) (pg/ml, x̄ ± s)	0.293	0.067	**−**0.029	0.857	**−**0.077	0.636	**−**0.218	0.178	0.066	0.686	0.096	0.567
T-tau (pg/ml, x̄ ± s)	0.205	0.198	0.011	0.945	**−**0.089	0.582	**−**0.105	0.512	0.083	0.605	**−**0.074	0.648

**P < 0.01. The bold values are statistically significant.

## Discussion

### Frequency of Alzheimer’s Disease With Hearing Loss

Currently, there is no study on the frequency of HL in AD patients. In this study, the frequency of AD-HL was 67.69%, demonstrating that HL is very common in AD patients. HL is one of the risk factors of AD that can be intervened. Therefore, evaluation of auditory function should be routinely performed, and HL should be intervened as early as possible for AD patients (Alzheimer’s Disease International [ADI], 2020). It is very necessary to conduct an in-depth exploration on the clinical features and related mechanisms of AD-HL, providing clinical evidence for finding new targets for intervention and slowing down the progression of the disease.

### Demographic Variables of Alzheimer’s Disease With No Hearing Loss and Alzheimer’s Disease With Hearing Loss Groups

Demographic variables, including sex, age, age of onset, disease duration, and years of education in AD-nHL and AD-HL groups were compared. The results showed no significant differences in the abovementioned demographic variables between the two groups, indicating that the following variables investigated in this study was comparable (Table 1).

### Cognitive Function, Neuropsychiatry Symptoms, and Activities of Daily Living in Alzheimer’s Disease With No Hearing Loss and Alzheimer’s Disease With Hearing Loss Groups

MMSE and MoCA scales are widely used for evaluating the overall cognitive function. In this study, the overall cognitive function was severely impaired in AD-HL group compared with AD-nHL group (Table 2). The score of each cognitive domain was then analyzed.

HL drastically compromised immediate memory and overall auditory memory for AD patients, and prevented AD patients from adopting strategies for memory process, such as classification, and did great harm to the ability of recognition (Tables 2, 3). However, HL exerted no remarkable influence on the delayed recall memory. In the mouse model of auditory deprivation, there were robust microglial activation and oxidative stress, which prominently elicited damage to hippocampal neurogenesis and caused subsequent impairment of immediate memory and learning (Kim et al., 2020; Kurioka et al., 2021). Hence, HL might aggravate memory impairment by suppressing hippocampal neurons *via* neuroinflammation and oxidative stress as well as AD pathology.

In this study, HL greatly contributed to the serious impairment of language function for patients with AD (Tables 2, 3). Language function was associated with a posterior section of superior and middle temporal gyrus (Yi et al., 2019) and left triangularis in frontal lobe and superior temporal lobe (Obler et al., 2010), which were close to the auditory cortex. Particularly, superior temporal gyrus combined hearing with speech due to its close connection with auditory cortex (Bernstein and Liebenthal, 2014). Therefore, HL might cause damage to language function due to the fact that their correspondent regions in brain were anatomically adjacent.

In this investigation, HL significantly propagated deficit of attention for patients with AD (Tables 2, 3). A previous investigation found that adults with attention deficit or hyperactivity disorders had worse performances in the tests of visual and auditory attention (Taitelbaum-Swead et al., 2019). It might be speculated that more cognitive resources were dedicated to auditory processing under the condition of HL, resulting in the depletion of the resources for multiple cognitive domains, including attention (Lin et al., 2013).

In this exploration, AD-HL group had worse performances in the tests of executive function than AD-nHL group (Tables 2, 3). It was reported that HL and concomitant impairment in auditory cortex increased the recruitment of executive function as well as short-term memory to aid speech perception (Wong et al., 2014), elucidating a redistribution of cognitive resources in the case of HL. It could be speculated that short-term reallocation of cognitive resources might improve performance of cognitive function as a compensation for HL, however, the levels of cognitive domains might decline as a result of exhaustion of overall cognitive resources, as the duration of HL was prolonged.

The impairment of visuospatial function is usually observed in the middle and late stages of AD. Here, patients recruited were in all stages of AD, and those who were in the early stage of the disease might have no visuospatial dysfunction, which might explain that AD-HL and AD-nHL groups were not different in the visuospatial function (Tables 2, 3). We will conduct an in-depth investigation including patients in different stages of AD in the future.

In this study, no significant differences of neuropsychiatric symptoms were observed between AD-HL and AD-nHL groups (Tables 2, 3). There are a body of neuropsychiatric symptoms of AD, among which, some occur in the early stage, like depression and anxiety, and some occur in the late stage, like hallucination and delusion. Patients in all stages recruited might account for the indiscrimination of neuropsychiatric symptoms between the two groups.

The current study showed that AD-HL group had a significantly compromised ADL (Tables 2, 3). It might be because that plenty of cognitive resources were dedicated to the auditory processing, and thus induced severe impairment of cognitive function, which eventually caused poor ADL.

### Alzheimer’s Disease With Hearing Loss and Neuropathological Biomarkers of Alzheimer’s Disease

Increasing studies revealed that AD pathology was relevant to HL. P-tau expression in hippocampus of mice with HL was significantly elevated (Omata et al., 2016). Simultaneously expressed Aβ_1–42_ and tau exerted an obvious synergistic effect, contributing to severe hearing defect (Braak and Braak, 1991). In the rare human studies, it was found that the levels of P-tau (T181) and T-tau but not Aβ_1–42_ level in CSF were significantly elevated in the HL individuals (Park et al., 2018), indicating that tau pathology played a more important role on HL than Aβ did in humans.

In this study, tau pathology indicated by the elevation of P-tau (S199) was highly associated with HL in patients with AD (Tables 4, 5). In AD, NFTs with the major component of P-tau started from layer II of entorhinal cortex, and finally arrived to hippocampus and neocortex (Braak and Braak, 1991). Although entorhinal cortex and hippocampus were mainly related to memory, they still had wide connections with auditory processing regions (Chen et al., 2013; Aronov et al., 2017; Ahmed et al., 2020). It was observed that HL increased tau phosphorylation *via* intensified neuroinflammation in mice/rat hippocampus (Cui et al., 2012; Shen et al., 2021). P-tau accumulation promoted neuroinflammation and oxidative stress in brain neurons (Alavi Naini and Soussi-Yanicostas, 2015).

Pathological tau conformers were transferred among cells through several ways and multiple mechanisms (Guo and Lee, 2014). Hence, we supposed that HL might increase deposition of P-tau, which was then spread from cognitive to auditory regions in brain, eventually aggravating the hearing dysfunction.

HL patients need and use up more cognitive reserve for auditory processing. The depletion of cognitive reserve was more vulnerable to AD pathology (Scarmeas and Stern, 2003). Because it is difficult to quantify cognitive reserve, the structural and neurobiological changes of cognitive reserve have not been elucidated so far. However, cognitive reserve and abnormal tau, including P-tau, led to the reduced synapses and shrunk dendrites (Guerrero-Muñoz et al., 2015). Additionally, HL led to social isolation due to the difficulties in communication, and thus served as a critical risk factor of AD (Wilson et al., 2007; Shankar et al., 2013; Boss et al., 2015). In a mouse experiment, isolation promoted AD pathology through neuroinflammation and oxidative stress (Schiavone et al., 2009; Huang et al., 2015; Liu et al., 2017), which might be the potential mechanism of social isolation relevant AD.

This study found that AD-HL was significantly related to the elevated P-tau (S199), but not declined Aβ, indicating that HL was more likely to play a significant role on the progression of AD reflected by the accumulation of P-tau (S199) formation, rather than in the early stage of AD indicated by the formation of Aβ. Thus, early intervention of HL might decrease the progression of tau pathology and thus prevent deterioration of AD.

This study failed to find differences in other forms of P-tau between AD-HL and AD-nHL groups, suggesting that P-tau (S199) might be the important neuropathological biomarker indicating AD aggravation by HL. The detailed mechanisms need to be investigated in the future.

### Relationship Among Neuropathological Biomarkers of Alzheimer’s Disease, Blood-Brain Barrier Factors, and Alzheimer’s Disease With Hearing Loss

In this study, HL reflected by PTA threshold was not only significantly correlated with AD neuropathological biomarker of P-tau (S199) level in CSF (Tables 4, 5), but also with BBB impairment reflected by the elevated MMP-3 level in CSF from AD patients (Tables 6, 7). In AD-HL patients, a further analysis suggested that P-tau (S199) level was significantly and positively correlated with the levels of MMP-2 and MMP-3 (Table 8).

MMPs was a multigene family of proteinases that played pivotal roles on the disrupted integrity of BBB (Lakhan et al., 2013) *via* digesting proteins of tight junction and basement membrane. Blood-labyrinth barrier and BBB had similarity in structure; thus, MMPs was inferred to cause damages to both of them. Previous studies in guinea pigs found that the levels of MMP-2 and MMP-9 in healthy vascularis were markedly increased after noise exposure, causing damage to tight junctional proteins, and compromising the instability of cochlear blood-labyrinth barrier (Wu et al., 2017). Accordingly, homeostasis of internal environment of auditory pathway was significantly compromised by MMPs.

MMP-2 and P-tau were co-localized in neurofibrillary tangles and dystrophic neurites under confocal microscopy (Terni and Ferrer, 2015). Furthermore, P-tau stimulated the expression of MMP-2 (Terni and Ferrer, 2015), accordingly, MMP-2 level might be elevated as P-tau level increased in brain. A previous study showed that, at early Braak stage of AD pathology, entorhinal cortex with increased MMP-2 had a wide connection with auditory pathway and cortex (Terni and Ferrer, 2015), proving more direct evidence of close relationships among AD pathology, MMP-2, and AD-HL.

MMP-3 was overexpressed in astrocytes and neurons exposed to Aβ, eliciting microglial activation, and activated microglia in turn propagated accumulations of Aβ and P-tau, which continuously precipitated microglial activation and intensified neuroinflammatory cascade event (Vasto et al., 2007). Thus, AD pathology might trigger the elevation of MMP through neuroinflammation. Moreover, AD and HL, as aging diseases, shared the similar mechanisms of neuroinflammation and oxidative stress, which precipitated P-tau deposition and MMP-3 expression, and both of them in turn promoted oxidative stress and neuroinflammation, producing plenty of free radicals and neuroinflammatory factors (Kim and Hwang, 2011). Particularly, advanced glycation end product, serving as an important initiator of oxidative stress and neuroinflammation, was significantly elevated in both AD and HL patients (Kim and Hwang, 2011; Niihata et al., 2018). The large amount of toxic neuroinflammatory factors and free radicals produced might transfer to the periphery through the disrupted BBB, and thus cause damage to peripheral auditory system and induce deterioration of HL in AD patients.

Socially isolated rats suffered from robust oxidative stress in brain (Schiavone et al., 2009; Colaianna et al., 2013). In a rat experiment, numerous generated neuroinflammatory factors and free radicals mediated BBB disruption through MMPs, such as MMP-2, MMP-3, and MMP-9 (Lehner et al., 2011). AD-HL patients were more prone to loneliness and social isolation, which intensified neuroinflammation and oxidative stress (Li and Xia, 2020), and therefore might elevate the levels of MMPs, like MMP-3, which was observed in this study.

The above findings established the relationships among P-tau (S199), MMP-2/MMP-3 and AD-HL. The mutual promotion between P-tau (S199) and MMP-2/MMP-3 might explain disease progression in AD patients with HL.

This study failed to find differences in other BBB factors between AD-HL and AD-nHL groups, suggesting that MMP-2/MMP-3 might be the important factors indicating BBB damage aggravated by HL in AD patients. The detailed mechanisms need to be studied in the future.

This research was a single-center study, and the results might be biased and need to be interpreted with caution. A multicenter study will be conducted in the future.

In summary, AD patients have a high frequency of HL. AD-HL patients have severely compromised overall cognitive function and multiple cognitive domains of memory, language, attention and executive function, and ADL. The potential mechanisms of AD-HL may involve the elevations of AD pathological biomarker of P-tau (S199) and BBB factor of MMP-3, and the close correlations between P-tau (S199) and MMP-2/MMP-3 in CSF. Findings from this investigation highly suggest that early evaluation of HL is very pivotal to delay AD progression, and cast a new light for drug development by inhibiting neuropathological biomarkers of AD and protecting BBB in the future.

## Data Availability Statement

The raw data supporting the conclusions of this article will be made available by the authors, without undue reservation.

## Ethics Statement

The studies involving human participants were reviewed and approved by Review Board of Beijing Tiantan Hospital, Capital Medical University. The patients/participants provided their written informed consent to participate in this study.

## Author Contributions

Wei-jiaoZ drafted the manuscript, carried out the analysis of data, accepted responsibility for the conduct of the research, final approval for the research, and performed the acquisition of data and the statistical analysis. D-NL, T-HL, PG, and X-MW carried out the analysis of data, accepted responsibility for the conduct of the research, and provided final approval. M-YH, Y-NZ, Wen-jingZ, D-ML, and Wei-jiaZ carried out the acquisition of data, accepted responsibility for the conduct of the research, and provided final approval. J-HL and H-YG drafted the manuscript, accepted responsibility for the conduct of the research, and provided final approval. WZ prepared the study design, carried out the analysis of data, accepted responsibility for the conduct of the research, provided final approval, performed the acquisition of data, the statistical analysis, and study supervision. All authors contributed to the article and approved the submitted version.

## Conflict of Interest

The authors declare that the research was conducted in the absence of any commercial or financial relationships that could be construed as a potential conflict of interest. The handling editor JL, declared a shared parent affiliation with the authors at the time of the review.

## Publisher’s Note

All claims expressed in this article are solely those of the authors and do not necessarily represent those of their affiliated organizations, or those of the publisher, the editors and the reviewers. Any product that may be evaluated in this article, or claim that may be made by its manufacturer, is not guaranteed or endorsed by the publisher.
